# Pleiotropic Effect of Hormone Insulin-Like Growth Factor-I in Immune Response and Pathogenesis in Leishmaniases

**DOI:** 10.1155/2021/6614475

**Published:** 2021-05-04

**Authors:** Luiza C. Reis, Eduardo Milton Ramos-Sanchez, Fernanda N. Araujo, Ariane F. Leal, Christiane Y. Ozaki, Orlando R. Sevillano, Bernardina A. Uscata, Hiro Goto

**Affiliations:** ^1^Instituto de Medicina Tropical de São Paulo, Faculdade de Medicina, Universidade de São Paulo (IMTSP-USP), São Paulo, Brazil; ^2^Departamento de Salud Publica, Facultad de Ciencias de La Salud, Universidad Nacional Toribio Rodriguez de Mendoza de Amazonas, Chachapoyas, Peru; ^3^Departamento de Medicina Preventiva, Faculdade de Medicina, Universidade de São Paulo, São Paulo, Brazil

## Abstract

Leishmaniases are diseases caused by several *Leishmania* species, and many factors contribute to the development of the infection. Because the adaptive immune response does not fully explain the outcome of *Leishmania* infection and considering that the initial events are crucial in the establishment of the infection, we investigated one of the growth factors, the insulin-like growth factor-I (IGF-I), found in circulation and produced by different cells including macrophages and present in the skin where the parasite is inoculated. Here, we review the role of IGF-I in leishmaniasis experimental models and human patients. IGF-I induces the growth of different *Leishmania* species in vitro and alters the disease outcome increasing the parasite load and lesion size, especially in *L. major-* and *L. amazonensis-*infected mouse leishmaniasis. IGF-I affects the parasite interacting with the IGF-I receptor present on *Leishmania*. During *Leishmania*-macrophage interaction, IGF-I acts on the arginine metabolic pathway, resulting in polyamine production both in macrophages and *Leishmania*. IGF-I and cytokines interact with reciprocal influences on their expression. IL-4 is a hallmark of susceptibility to *L. major* in murine leishmaniasis, but we observed that IGF-I operates astoundingly as an effector element of the IL-4. Approaching human leishmaniasis, patients with mucosal, disseminated, and visceral diseases presented surprisingly low IGF-I serum levels, suggesting diverse effects than parasite growth. We observed that low IGF-I levels might contribute to the inflammatory response persistence and delayed lesion healing in human cutaneous leishmaniasis and the anemia development in visceral leishmaniasis. We must highlight the complexity of infection revealed depending on the *Leishmania* species and the parasite's developmental stages. Because IGF-I exerts pleiotropic effects on the biology of interaction and disease pathogenesis, IGF-I turns up as an attractive tool to explore biological and pathogenic processes underlying infection development. IGF-I pleiotropic effects open further the possibility of approaching IGF-I as a therapeutical target.

## 1. Introduction

Leishmaniases are considered neglected tropical diseases caused by parasites of the order Kinetoplastida, family Trypanosomatidae, and genus *Leishmania*, affecting one million people globally each year and endemic in 98 countries. The transmission occurs through a sandfly bite that inoculates promastigotes into the skin, which transform into amastigotes and proliferate within phagocytic mononuclear cells. The infection can be asymptomatic or symptomatic, leading to a wide spectrum of clinical manifestations ranging from localized, disseminated, or diffuse cutaneous lesions or mucosal lesions to viscera involvement such as the liver and spleen [1]. The establishment of these different clinical forms depends on the parasite's species and on the vector, in addition to the epidemiological characteristics and genetic and immunological constitution of the host [2], where both innate and adaptive immune responses can drive the development or control of infection.

The beginning of our studies on insulin-like growth factors on *Leishmania* and leishmaniases backs to the nineties when the main focus on leishmaniasis research was the cell-mediated immune response. At the end of the eighties, T helper 1 (Th1) was related to resistance and T helper 2 (Th2) to susceptibility to *Leishmania major* infection in studies using inbred mouse strains. The susceptibility was attributed to early Th2 cell activation present in BALB/c mice and the resistance to Th1 cells in C57BL/6 mice by the infection. Subsequent studies using different approaches as cytokine neutralization, specific cell response induction, or inhibition of the immune system's elements through molecular deletions confirmed this view, but others pointed flaws in this model. Similar studies using other *Leishmania* species also showed that the adaptive immune response does not explain the infection's resistance and susceptibility profiles. Further, human leishmaniases' pathogenesis cannot be explained only based on resistance and susceptibility to parasite growth.

In this review, we initially present some data related to the involvement of adaptive immune response on *Leishmania major and Leishmania donovani*/*Leishmania infantum* infections that do not fully clarify infection development to justify the focus here on nonspecific factors. Then, we present data including studies on the innate immune response that points out our research on the IGF-I growth factor as an element that may contribute to *Leishmania* infection.

## 2. Adaptive Immune Response in Leishmaniases: Flaws

### 2.1. *Leishmania major*

The experimental model using *L.* (*L.*) *major* has been used to characterize the immune response on resistance or susceptibility to infection related to the Th1 and Th2 cell activation, respectively [3]. The role of cytokines in the Th1 and Th2 paradigm has been questioned by results suggesting that the resistance and susceptibility regulation are much more complex, involving cytokine production and other factors [4–9]. Studies have demonstrated that the interferon- (IFN-) *γ* and interleukin- (IL-) 4 production are similar in susceptible and resistant mice in the early stage of infection [3, 10, 11]. Further, IL-4 production in resistant mice does not alter the evolution towards progressive disease, similarly seen in C3H mice treated with IL-4 or anti-IL-12 at the beginning of infection. These mice presented a solid but transient increase in the IL-4 level, with no change in their resistant phenotype [12–14]. Another study showed that the transfer of BALB/c T cells with high IL-4 expression to genetically resistant chimeric mice having a C57BL/6 background did not result in susceptibility [15]. These questions on the Th1 and Th2 paradigm in leishmaniasis also become evident in infections caused by other *Leishmania* species.

### 2.2. *Leishmania donovani*/*Leishmania infantum*

In contrast to the *L. major*-infected mouse model's immune response very much scrutinized, the immune mechanisms in experimental visceral leishmaniasis (VL) are less explored. Resistance in VL involves CD4^+^ and CD8^+^ T cells, IL-2, IFN-*γ*, and IL-12, the latter in an IFN-*γ*-independent mechanism and linked to transforming growth factor *β* (TGF-*β*) production. Susceptibility involves IL-10 but not IL-4 and B cells [16]. Studies on *L. donovani* and *L. infantum* infections in mice and humans [17–20] suggest that control of infection was independent of the Th1 and Th2 cytokine differential production (IFN-*γ*/IL-4 balance) [20]. The resistance to *L. donovani* and induction of granuloma formation are dependent on the generation of an IFN-*γ* response by both CD4^+^ and CD8^+^ T cells. IFN-*γ* activates macrophages to produce antimicrobial reactive nitrogen and oxygen intermediates [21], also important in driving granuloma maturation. Most *L. donovani*-infected mouse strains control infection spontaneously, becoming immune for subsequent infections. In these immune animals, upon reinfection, the elements involved in resistance are different, i.e., CD8^+^ T cells and IL-2 [16]. These experimental VL findings do not contribute substantially to understand active human VL where the disease is progressive and lethal if not treated.

In active human VL, the patients present fever, hepatosplenomegaly, hypergammaglobulinemia, pancytopenia, and significant weight loss [1]. Most of the studies focus on the suppression of the T cell responses [22]. Immunosuppression is characterized as *Leishmania* antigen-specific, where T cells, Th2 cells, and adherent antigen-presenting cells are involved [16]. Cytotoxic T-lymphocyte-associated protein 4 (CTLA-4) and programmed cell death protein 1 (PD-1) are negative regulators of T cells and are expressed on exhausted or anergic T cells in active infection, taking part in immunosuppression. The evoked mechanism is the induction of an increased level of TGF-*β* and apoptosis of CD4^+^ T cells and inhibition of macrophage apoptosis by *Leishmania* infection [23]. T cell apoptosis, mainly CD4^+^ T cells, accompanied by a significant decrease in IL-2 and IFN-*γ* secretion and unaltered IL-4 secretion, was observed during *L. donovani* infection and was also related to immunosuppression [16]. Other immunosuppressive mechanisms established during VL may be mediated through regulatory T cells, secreting regulatory cytokines like IL-10 and TGF-*β* and expressing inhibitory molecules such as CTLA-4 and IL-35 [23].

In contrast to the studies on immunosuppression, other data suggest intense immune activation in active VL [24]. Studies on peripheral blood mononuclear cells raised data that suggest immunosuppression. However, findings in lymphoid organ samples such as bone marrow and spleen show high expression of tumor necrosis factor *α* (TNF-*α*) and IFN-*γ* mRNA levels and high IFN-*γ* serum level as a consequence [25, 26]. A study showing exhaustion of the immune system [27] reinforces this view. All these studies focusing on the balance between elements of adaptive immune mechanisms are aimed at explaining the active disease's development. Still, no clear answer was achieved, demanding the investigation on other components as the nonspecific factors.

## 3. Growth Factors in the Innate Response in Leishmaniases

As the adaptive immune response does not fully explain the control or development of *Leishmania* infection and the initial events that occur immediately after the vector's promastigote inoculation are crucial, we chose to investigate growth factors present on the inoculation site. Once inoculated into the skin, the parasites immediately encounter innate immune elements, including growth factors such as TGF-*β* and granulocyte-macrophage colony-stimulating factor (GM-CSF).

The role of TGF-*β* as an important immune regulator in leishmaniasis has been demonstrated in vitro [28–31] and in vivo [32–34]. TGF-*β* can inhibit macrophage activation through the blockage of nitric oxide (NO) production, leading to an increase in the parasitic load. Further, a synergistic effect between TGF-*β* and EBI-3 (Epstein Barr virus-induced gene 3 or IL-27*β*) on suppression of BALB/c mouse immune response infected by *L. donovani* was observed [35]. Interestingly, TGF-*β* homolog found in *Lutzomyia longipalpis* was suggested to contribute to the *L. infantum* survival within the vector [36].

In addition to TGF-*β*, GM-CSF can interact with *L. amazonensis* promastigotes, promoting their growth and protecting them from death by thermal shock [37, 38]. In accordance, the rGM-CSF (recombinant GM-CSF) treatment in *L. major*-infected BALB/c mice promoted an enlargement in lesions and increased parasite load, as well as *L. major*-infected macrophages incubated with rGM-CSF presented an increased parasite load [39]. Further, the GM-CSF was used topically during Miltefosine treatment in American tegumentary leishmaniasis without success [40, 41]. In a multiomic study in cutaneous leishmaniasis patients, diminished concentrations of GM-CSF, IFN-*α*2, IL-6, and IL-3 and increased eotaxin levels were related to treatment failure [42]. However, other studies show opposite results; parasites are eliminated in *L. tropica-*, *L. mexicana*-, and *L. donovani-*infected macrophages upon activation with GM-CSF [43–45]. Moreover, mice with a null mutation in the gene for the beta common (*β*c) chain of the receptors for GM-CSF, IL-3, and IL-5 infected with *L. major* presented resistance to infection [46].

In this context, we have started studying another growth factor, the insulin-like growth factor-I (IGF-I). Its role in *Leishmania* infection was considered because it is produced by different cell types, including macrophages that harbor the parasites. Also, it is present in the skin, where the parasite initiates the infection [47].

The IGF-I is a hormone that presents a molecular structure relatively similar to insulin, being produced primarily by the liver under the control of growth hormone (GH). As a polypeptide phylogenetically well preserved, the IGF-I presents a molecular mass of approximately 7.5 kDa. It is present in the circulation bound to a complex of carrier proteins called insulin-like growth factor-binding proteins (IGFBPs). Since its affinity for IGFBPs is greater than for its receptor, in the extracellular environment, most IGFs bind to IGFBP, of which IGFBP-3 is the most abundant in human serum [48]. As mentioned before, different cell types produce IGF-I, including macrophages that produce and harbor the IGF-I with 26 kDa that will be cleaved in a 7.5 kDa molecule to be secreted. IGF-I exhibits pleiotropic properties, including the ability to promote cellular proliferation, differentiation, nutrient transport, energy storage, gene transcription, protein synthesis, modulation of the immune response and inflammation, and epigenetic modifications [47, 49, 50]. Of note, IGF-I can trigger and/or modulate more than 200 genes depending on cell types, tissues, development stages, among others [51, 52]. In addition to insulin, the IGF-II is another molecule that presents considerable similarity with IGF-I, and its effect was observed on the stimulation of *Giardia lamblia* trophozoite growth [53]. Despite the substantial similarity between IGF-I and IGF-II, only IGF-I presented effect on *Leishmania* infection and proliferation [54–56]. The same was observed on the development of infection caused by other pathogens, like *Mycobacterium leprae*, *Schistosoma mansoni*, and *Schistosoma japonicum* [57, 58].

The IGF-I exerts its biological effects binding to its receptor (IGF-IR), which is present in several cell types, mainly in macrophages, activating the intracellular signaling cascade. The phosphoinositide-3 kinase/protein kinase-B (PI3K/AKT) and the mitogen-activated protein kinase (Ras/MAPK/ERK) are two main pathways activated by IGF-I. Their stimulation may also occur by binding to the insulin receptor (IR) when its free form is present in excess. Since IGF-IR and IR are highly homologous tyrosine kinase receptors sharing many signaling pathway components and inducing insulin receptor substrates 1 and *2* (IRS1/2) in addition to AKT and MAPK phosphorylation [59–61], the IGF-I binding to IR can result in a signaling cascade comparable to the one triggered by the IGF-IR, generating similar effects.

It has been demonstrated that some physiological processes are controlled by the immune and endocrine systems reciprocally, through cytokine and hormone-regulated actions [62–64]. Regarding IGF-I, some factors such as cytokines can regulate its expression in macrophages. Macrophage stimulation, in vitro, by IFN-*γ* results in a decrease and by IL-4 and IL-13 in an increase of IGF-I expression [65–67]. On the other hand, IGF-I can also regulate cytokine production. Phytohemagglutinin- (PHA-) stimulated human peripheral blood mononuclear cells (PBMC) in the IGF-I presence showed an increase in IL-10 and IL-4 and decrease in IFN-*γ* secretion. In accordance, the IL-10 mRNA level increased, as well as IL-10 secretion in PBMC-derived T cells under the same conditions [68]. Further data showing expression of IGF-I receptor expression upon T cell activation or modulation of adaptive immune elements by IGF-I [69–72] suggest an important role of IGF-I in immunity.

This review will present the IGF-I as an active participant both in experimental infection by *Leishmania spp* and human leishmaniasis.

## 4. IGF-I in Experimental Leishmaniasis

### 4.1. Effect of IGF-I on *Leishmania* Promastigotes and Amastigotes

The IGF-I likely interacts with *Leishmania* immediately after its inoculation into the host's skin and after being phagocytized by macrophages. For years, we studied the participation of this growth factor directly on *Leishmania spp* promastigotes and amastigotes in experimental in vitro and in vivo infection and human infection.

**Figure 1:**
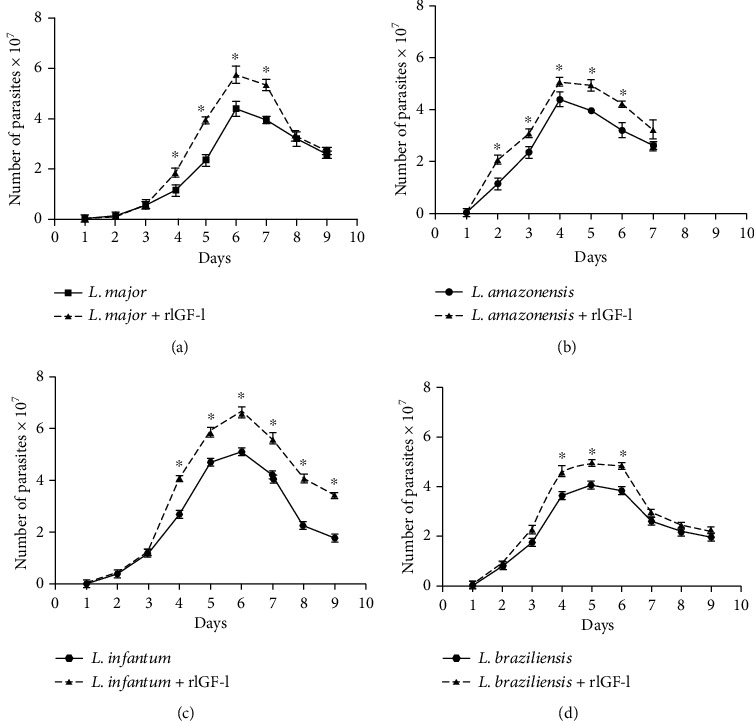
Growth curves of *Leishmania spp* promastigotes upon IGF-I stimulus. *L. major*, *L. amazonensis*, *L. infantum*, and *L. braziliensis* promastigotes (5 × 10^5^/mL) were cultured in 199 medium (Cultilab, Brazil) and Schneider's Insect medium (Sigma-Aldrich, USA), respectively, supplemented with 5% heat-inactivated fetal calf serum (FCS) (Cultilab, Brazil) at 26°C, with or without 50 ng/mL IGF-I (rIGF-I, R&D Systems, USA). The growth of parasites was monitored by daily counting, for 10 days, in a Neubauer chamber, and the results are presented as the number of parasites × 10^7^/mL (mean ± standard deviation) from three independent cultures with or without 50 ng/mL IGF-I. ^∗^*p* < 0.05 (one-way ANOVA) compared with the culture without IGF-I (adapted from Reis et al. [65]).

We initially evaluated the IGF-I effect on promastigotes and axenic amastigotes of different *Leishmania* species by adding extrinsic IGF-I (i.e., recombinant human IGF-I) in physiological concentrations [54–56]. Analyzing the IGF-I effect throughout the promastigote growth curves of each species, we observed increased proliferation of the parasites in the presence of IGF-I. Its effect was more evident when they reached the stationary growth phase (Figure 1). Thus, the promastigotes' response to extrinsic IGF-I suggests that their contact with the host's IGF-I when inoculated into the skin can substantially affect them at the initial stage of infection.

Subsequently, binding of IGF-I was shown to induce phosphorylation of tyrosine (185 kDa and 60 to 40 kDa proteins) and serine-threonine residues (110 kDa and 120 and 95 kDa proteins) in promastigotes and axenic amastigotes, in a stage-specific effect [73]. When analyzing the interaction between IGF-I and parasites, it was shown that IGF-I binds specifically to a single-site putative receptor at the parasite membrane. The receptor is a monomeric glycoprotein with a molecular mass of 65 kDa and is antigenically related to the *α* chain of human type 1 IGF-I receptor [74]. This specific IGF-I receptor found on the surface of *Leishmania* promastigotes and amastigotes differs considerably from those found on mammalian cells. In human cells, the IGF-I receptor is constituted by two alpha and two beta chains with a molecular mass of 135 and 93 kDa, respectively [74, 75]. Upon IGF-I stimulation, the receptor goes through autophosphorylation on tyrosine residues, activating the signaling pathway. Activation of the IGF-I receptor on *Leishmania* also leads to the phosphorylation of a 185 kDa molecule that is homologous to the insulin receptor substrate present in human cells, the IRS-1. IRS-1 is a critical adapter protein involved in IGF-I signaling and is considered a docking protein, playing a central role in the intracellular signaling network [74, 76]. We may speculate that the receptor functions as part of an array of adaptive responses developed by the parasite to survive within the host. Thus, it may be considered a possible vaccine target. In fact, it has been approached in schistosomiasis since the IR in *Schistosoma mansoni* and *S. japonicum* were discovered. These worms like *Leishmania* use the host's hormones and nutrients for their development. Their insulin receptors have been studied as a vaccine target in animal models to prevent transmission [57].

### 4.2. Effect of IGF-I on *Leishmania*-Macrophage Interaction

Moving to in vivo studies using a cutaneous leishmaniasis model in BALB/c mice, we have shown that the preincubation of *L. amazonensis* promastigotes with IGF-I promotes a significant increase in the footpad lesion size, 21 days postinfection. We observed an increase in the number of parasites in the lesion accompanied by an inflammatory infiltrate. These results suggest that IGF-I has a significant role in the innate immune response during the infection, favoring the parasite growth within macrophages [77].

Since IGF-I favors the macrophage's parasite growth, this growth factor probably affects the macrophage's metabolic machinery. The macrophages are key cells in the establishment of *Leishmania* infection. Depending on the cells' activation stimuli, *Leishmania* infection's development will result in progression or cure. One of the mechanisms involved in these processes is the L-arginine metabolic pathway. The L-arginine enters the cells from the extracellular milieu by the cationic amino acid transporter 2 (CAT-2B), a member of the classical amino acid cationic transporter system y+ (SLC7) [78]. When this amino acid is oxidized by the nitric oxide synthase 2 (NOS2), it generates nitric oxide (NO), one of the main leishmanicidal elements. However, when the enzyme arginase hydrolyzes L-arginine, polyamines are generated, promoting *Leishmania* proliferation [79–81]. Thus, we addressed the study of extrinsic IGF-I's effect on the parasite-macrophage interaction in vitro, evaluating the L-arginine metabolic pathway in macrophages' infection with *L. amazonensis*.

**Figure 2:**
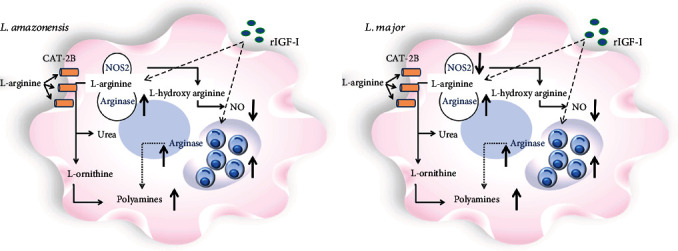
Scheme of the effect of extrinsic IGF-I (rIGF-I) in the L-arginine metabolic pathway activation in macrophages infected by *L. amazonensis* and *L. major*. In RAW 264.7 cells or BALB/c mouse peritoneal macrophages infected with *L. amazonensis* or *L. major* promastigotes and stimulated with 50 ng/mL recombinant IGF-I (rIGF-I, R&D Systems, USA), the parasitism, arginase mRNA expression, and arginase activity, nitric oxide synthase 2 (NOS2) mRNA expression, and nitric oxide production (Griess reaction) were evaluated. Extrinsic IGF-I induced an increase in arginase expression and arginase activity in both parasites and macrophages, decreased the production of NO, and increased the parasitism in *L. amazonensis*- and *L. major*-infected cells, comparably.

In this approach, we observed that IGF-I favored parasite growth in *L. amazonensis*-infected macrophages. It occurred through an increase in arginase mRNA expression and arginase activity in both parasites and macrophages and decreased the production of NO by macrophages [55]. As each species of *Leishmania* behaves differently, we analyzed the role of extrinsic IGF-I on *L. major*. Similarly to those results obtained with *L. amazonensis*, in *L. major*-infected macrophages, IGF-I favored the parasite proliferation within the macrophage inducing the arginase activation with an increase of arginase mRNA expression and arginase activity in both parasites and macrophages and a decrease in the *Nos2* mRNA expression and the production of NO by macrophages [65, 82] (Figure 2). These results showed the effect of extrinsic IGF-I on L-arginine metabolism leading to the parasite's proliferation within the macrophage.

It is worth mentioning that in *Leishmania* infection, the fate of host-parasite interaction depends on the *Leishmania* species involved. With this in mind, we evaluated the effect of IGF-I on the macrophage infection with other species, *L. infantum*, which causes visceral leishmaniasis (VL) and *L. braziliensis*, which is responsible for the cutaneous (CL), disseminated (DL), and mucosal (ML) forms of the disease.

**Figure 3:**
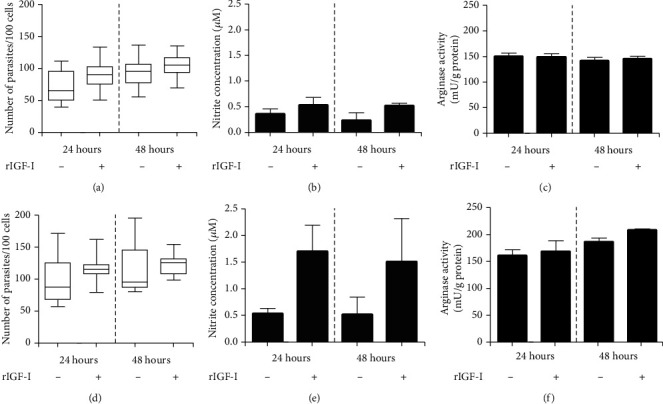
Parasitism and nitric oxide production in *L. infantum*-infected cells upon extrinsic IGF-I stimulus. THP-1 cells (a–c) or BALB/c mouse bone marrow-derived macrophages (d–f) were infected with *L. infantum* promastigotes and stimulated with recombinant IGF-I (rIGF-I, 50 ng/mL; R&D Systems, USA) for 24 and 48 hours. One representative experiment from three independent assays is shown. THP-1 cells were differentiated into macrophages with 20 ng/mL phorbol 12-myristate 13-acetate (PMA; Sigma-Aldrich, USA) for 24 hours. Then, the cells were washed and allowed to rest in a fresh medium for 48 hours before infection with *L. infantum* promastigotes. The parasitism (median number of parasites per 100 cells), nitric oxide production (Griess Reagent), and arginase activity (urea production) were determined after 24 and 48 hours of incubation.

For the evaluation of the IGF-I effect on *L. infantum* infection, we used the THP-1 human monocytic cell line and murine macrophages. In *L. infantum*-infected THP-1 cells upon IGF-I stimulus, the increase in parasitism was not evident, only a slight tendency, accompanied by an increase in NO production and no difference in arginase activity (Figures 3(a)–3(c)). We observed similar results in *L. infantum*-infected bone marrow-derived murine macrophages enquiring whether the cell type influences these results. We noted an increasing trend in parasitism, accompanied by increased NO production upon stimulation with IGF-I and no difference in arginase activity (Figures 3(d)–3(f)). Thus, unlike *L. major* and *L. amazonensis* infection, these data suggest that IGF-I does not significantly influence *L. infantum* amastigote proliferation. It may be related to the difference in the L-arginine metabolic pathway in this *Leishmania* species, where arginase does not seem essential for polyamine production. In studies with *L. donovani*, a related species that cause VL, arginase-deleted amastigotes survive within the cell without polyamine supplement but not promastigotes [83–85].

Further, in *L. infantum-*infected BALB/c and Swiss Webster mice, in the presence of spleen and liver cells producing high NO levels but with low arginase activity, the parasites continue multiplying within the host cells [86]. In vitro, *L. infantum* survives in the presence of high amounts of NO added in the culture medium [87]. Altogether, these data support the view that viscerotropic strains show differences in L-arginine metabolism.

As observed with other *Leishmania* species, the extrinsic IGF-I promoted *L. braziliensis* promastigote proliferation (Figure 1). However, this effect was not evident in *L. braziliensis* amastigotes within THP-1 cells upon stimulation with IGF-I. Analyzing the parasitism upon IGF-I stimulus in THP-1 cells infected with parasites isolated from patients presenting different clinical manifestations, CL, ML, and DL, no differences were observed. We noted only a slight tendency to increase and decrease parasitism in cells infected with parasites derived from ML and DL patients, respectively. We also investigated the involvement of IGF-I on arginase activation in both *L. braziliensis* promastigote-infected THP-1 cells. In promastigotes isolated from CL and DL patients, the arginase activity was increased after IGF-I stimulation, while in parasites isolated from ML patients, a decrease was observed. It is worth mentioning that the ML-derived parasites presented a higher arginase activity when compared with CL- and DL-derived parasites. Besides the alteration in arginase activity in promastigotes, no difference in arginase activity was detected in macrophages infected with those parasites derived from different disease manifestations [88, 89]. Besides, no differences were observed on NO production between the groups stimulated or not with IGF-I (personal communication). Macrophage metabolism in *L. braziliensis* derived from diverse clinical manifestations is poorly understood, demanding more studies on the role of IGF-I.

### 4.3. Effect of Macrophage Intrinsic IGF-I on Intracellular *Leishmania* Growth

**Figure 4:**
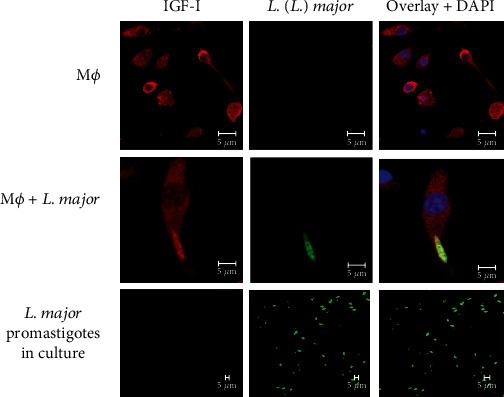
Detection of IGF-I within RAW 264.7 macrophages following infection with *Leishmania major* promastigotes. Colocalization of IGF-I and *Leishmania* was analyzed using immunofluorescence. Following a 24 h in vitro infection, cells were fixed in 4% paraformaldehyde (Sigma-Aldrich, USA), washed in 0.01 M phosphate-buffered saline, pH 7.2 (PBS), blocked for one hour with 2% bovine serum albumin (BSA; Sigma-Aldrich, USA) in PBS, and incubated overnight with monoclonal goat anti-mouse IGF-I antibody (1 : 75; R&D Systems, USA) and a polyclonal mouse anti-*Leishmania* antibody (1 : 400) [136]. Anti-goat IgG Alexa Fluor-546 (1 : 200, Invitrogen, USA—shown in red) and anti-mouse IgG Alexa Fluor-488 (1 : 400, Invitrogen, USA—shown in green) were used as secondary antibodies. 4,6-Diamidino-2-phenylindole (DAPI, Invitrogen, USA—shown in blue) was used to stain nuclei. Images were captured using a Leica LSM510 confocal microscope with a 63x objective and oil immersion (adapted from Reis et al. [82]).

As macrophages contain endogenous IGF-I in the cytoplasm, we decided to evaluate IGF-I's role produced by the macrophages (intrinsic IGF-I) in *Leishmania* infection. Under confocal microscopy, we showed that the intrinsic IGF-I interacts with intracellular *Leishmania* parasites [82] (Figure 4).

Since the interaction occurs between intrinsic IGF-I and intracellular parasites, we evaluate the parasitism upon inactivation of intrinsic IGF-I. Using a knockdown strategy, the *Igf-I* mRNA was silenced with IGF-I small interfering RNA (siRNA) in *L. major*-infected macrophages. In the siRNA-transfected group, we observed a significant decrease in parasitism, accompanied by decreased arginase mRNA expression and arginase activity in both parasites and macrophages and an increase in the *Nos-2* mRNA expression and NO production, when compared with the control group without siRNA transfection. This effect was reversed by the addition of recombinant IGF-I (rIGF-I), which induced an increase in the number of parasites and increased the levels of *Leishmania* arginase mRNA expression and arginase activity, accompanied by a decrease in the *Nos-2* mRNA expression and NO production [65].

**Figure 5:**
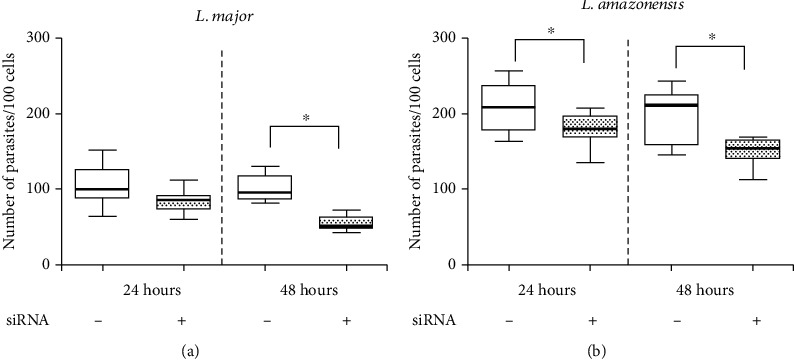
Parasitism in *L. major*- or *L. amazonensis*-infected macrophages upon IGF-I siRNA transfection. RAW 264.7 cells infected with *L. major* or *L. amazonensis* promastigotes transfected with or without 150 *μ*M IGF-I siRNA for 6 hours. The parasitism (median number of parasites per 100 cells) was evaluated after 24 and 48 hours. One representative experiment from three independent assays is shown. ^∗^*p* < 0.05 (ANOVA and Tukey's tests).

We observed similar results when the IGF-I silencing strategy was employed in *L. amazonensis-*infected macrophages. We observed a significant decrease in parasitism accompanied by an increase in the NO production in the groups treated with siRNA compared with the control without siRNA (Figure 5). In another study, macrophages from growth hormone (GH)/IGF-I-deficient individuals, due to the growth hormone-releasing hormone receptor gene mutation, were assayed in vitro for *L. amazonensis* infection. It was observed that the macrophages isolated from these individuals were less prone to infection than healthy control macrophages [90]. These data certainly confirmed the role of intrinsic IGF-I in intracellular parasite growth.

## 5. IGF-I and Cytokines

In *Leishmania* infection, the cytokines produced by macrophages and lymphocytes have an important participation in the interplay between parasite and the host involving IGF-I. In *L. amazonensis*-infected macrophages, the mechanisms leading to parasite growth upon IGF-I stimulus differed depending on the parasite life cycle stage used for infection. When cells were infected by promastigotes, upon IGF-I stimulus, this activation likely occurred through modulation of cytokine production, inducing a decrease in TNF-*α* and an increase in TGF-*β* and IFN-*γ* [55]. In cells infected by amastigotes, the mechanism was diverse. IGF-I induced phosphatidylserine exposure on the parasite surface that likely activated the macrophage arginase [91].

Other cytokines can act on the IGF-I expression like IFN-*γ* which promotes a reduction in the IGF-I expression, while IL-4 and IL-13 promote an increase in IGF-I expression [65–67]. These data suggest that IGF-I is involved in the development of the adaptive immune response in leishmaniasis.

The idea of immune-endocrine cross-talk has been described in other studies examining the roles of prolactin, GH, IGF-I, and thyroid-stimulating hormone in the development, maintenance, and function of the immune system, which in turn cause changes in the endocrine system [92–94]. The interaction between the endocrine and immune systems is somewhat expected, as they share several ligands and receptors in their signaling pathways [65].

Interactions between IGF-I and the immune system are complex, bidirectional, and not fully explained. IGF-I could modulate the inflammatory response and the activity of systemic inflammation [95]. Studies have indicated that chronic inflammation could suppress the IGF-I axis via several mechanisms such as downregulation of IGF-I receptors, disruption in the IGF-I signaling pathways, dysregulation of IGFBPs, reduced IGF bioavailability, and modified gene regulation through the changes in the microRNA expression [95, 96]. Proinflammatory cytokines such as IL-6, TNF-*α*, and IL-1*β* impair the activity of the IGF-I axis by dysregulation of its intracellular mediators, such as mitogen-activated protein kinase (MAPK)/extracellular signal-regulated kinases and PI3K [97]. IGF-I can reduce inflammation induced by oxidized low-density lipoprotein treatment by reducing high-mobility group box 1 (HMGB1) release, a potent stimulator of tissue damage and inflammation, after stimulation with pathogens or a factor passively released by necrotic cells, activating nuclear factor kappa B (NF-*κ*B) [98, 99]. In another study on postmyocardial infarction, IGF-I decreased myocardium cell apoptosis and inhibited gene expression and production of proinflammatory cytokines, such as TNF-*α*, IL-1*β*, and IL-6 [100]. Overall, most of the data appoint the effect of IGF-I on the innate inflammatory response.

In leishmaniasis, the specific immune response is well established in the *L. major*-infected mouse model, where Th1 and Th2 cytokines were, respectively, related to resistance and susceptibility to the infection. When Th1 cells are predominantly activated, the cytokines IL-2, IFN-*γ*, TNF-*β*, and IL-12 will be produced. Then, macrophages are activated and NOS2 induced, which metabolizes L-arginine, generating citrulline and NO associated with increased microbicidal activity. However, when Th2 cells are predominantly activated, mainly IL-4, IL-10, TGF-*β*, and IL-13 will be produced. Again, macrophages are activated alternatively, and arginase I expression and arginase activity are induced, leading to polyamine production that contributes to parasite proliferation [3].

When we analyzed IGF-I expression and the parasitism of *L. major* infection in vitro, macrophages stimulated with IFN-*γ* exhibited a reduction in the parasite load, accompanied by a parallel reduction in IGF-I expression and arginase activity and an increase in NO production. Further, IL-4 and IL-13 stimuli increased the parasitism, followed by a parallel increase in IGF-I expression and arginase activity and reduced NO production [65]. These data showing the similar effects of those cytokines on IGF-I expression and parasitism compelled us to explore this hormone's interference on the effects of cytokines during *Leishmania* infection.

As shown above, the Th1 and Th2 paradigm defined in the murine model with *L. major* infection has imperfections, and IL-4 related to susceptibility cannot be considered valid in any situation. Recently, in another *Leishmania* species, IL-4 was considered cytokine determining resistance in a *L. donovani*-infected BALB/c mouse [20]. Since the studies suggest that the susceptibility profile is not exclusively due to IL-4, and analyzing the signaling pathways of IL-4/IL-13 compared with the IGF-I pathway, we noticed shared components, suggesting that IGF-I and IL-4 may reciprocally interfere during *Leishmania* infection [65]. Thus, we proceeded with the study on the interference of IGF-I on IL-4 effect in *L. major*-macrophage interaction.

In *L. major*-infected macrophages stimulated with cytokines upon *Igf-I* mRNA expression silencing, the parasitism did not show the specific cytokine effect's expected result. Increased parasitism would be anticipated with IL-4 and IL-13 stimuli. However, they were utterly ineffective when the *Igf-I* mRNA was silenced. The effects of IL-4 and IL-13 on *Igf-I* mRNA-silenced cells were restored by the addition of rIGF-I in the culture, in a mechanism dependent on *Leishmania* arginase production [65].

**Figure 6:**
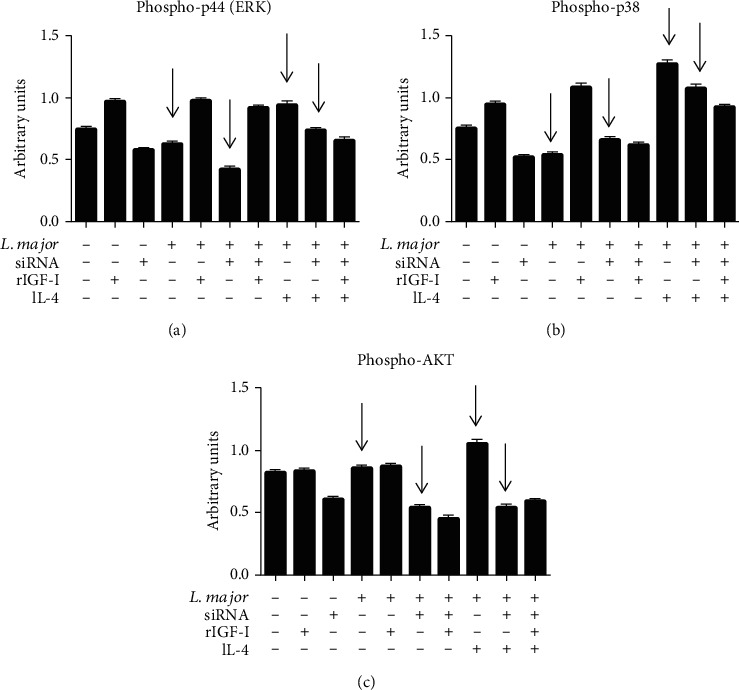
The effects of IGF-I siRNA and IL-4 on components of the IGF-I signaling pathways: levels of phosphorylated p44 (ERK), p38 (MAPK), and AKT proteins. *L. major* promastigote-infected or noninfected RAW 264.7 cells transfected with or without IGF-I siRNA were stimulated for 30 minutes with IL-4 (2 ng/mL; R&D Systems, EUA) and recombinant IGF-I (50 ng/mL; R&D Systems, EUA). Cells were lysed, the proteins were separated in 10% SDS-PAGE, and subsequently, a Western blotting was performed using anti-phospho-p44 (137F5, Cell Signaling Technology, USA), anti-phospho-p38 (D13E1, Cell Signaling Technology, USA), and anti-phospho-AKT (Ser473, Cell Signaling Technology, USA) antibodies. Protein bands corresponding to protein expression levels were submitted to a densitometric analysis (AlphaEaseFC™ software 3.2 beta version; Alpha Innotech Corporation, USA), and data are expressed in arbitrary units (adapted from Reis et al. [65]).

Our results showed that IGF-I is necessary for IL-4 to exert its effect on parasite proliferation in macrophages. IGF-I and the cytokines IL-4 and IL-13 share common components in their intracellular signaling pathways. IGF-I triggers MAPK (ERK) and PI3K pathways [59, 101], and IL-4 sequentially activates IRS-2 and the PI3K/Akt and Ras-MAPK pathways [61, 102]. We observed an increase in the levels of these phosphorylated proteins in all groups treated with IL-4. Upon *Igf-I* mRNA silencing, we observed a decrease in the expression of all phosphoproteins, and interestingly, IL-4 stimulation did not completely restore the decreased expression of phospho-p44 (ERK), phospho-p38 (MAPK), and phospho-AKT [65] (Figure 6). We thus considered IGF-I as the effector element for the IL-4 effect in promoting susceptibility in *L. major* infection.

**Figure 7:**
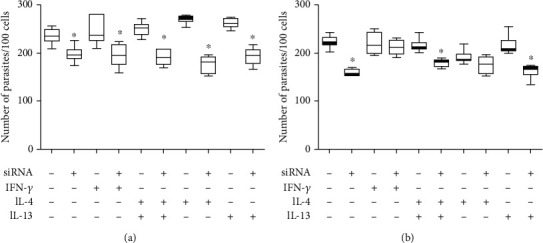
Parasitism in response to cytokine treatments and IGF-I siRNA transfection. Parasitism (median number of parasites per 100 cells) in *L. amazonensis*-infected RAW 264.7 cells transfected with or without 150 *μ*M IGF-I siRNA for 6 hours and then were stimulated with IFN-*γ* (200 U/mL; R&D Systems, EUA), IL-4 (2 ng/mL; R&D Systems, EUA), or IL-13 (5 ng/mL; R&D Systems, EUA) for 24 (a) and 48 (b) hours. One representative experiment from three independent assays is shown. ^∗^*p* < 0.05 (ANOVA and Tukey's tests).

The participation of both Th1 and Th2 cytokines in resistance and susceptibility was defined on *L. major*-infected murine model infection, but it may not work in other *Leishmania* species such as *L. amazonensis*. IFN-*γ* promotes parasite growth in *L. amazonensis* amastigote-infected macrophages [103]. In our study with *L. amazonensis*-infected macrophages, IFN-*γ* induced increased parasitism and NO production and decreased IGF-I expression, with no correlation with the parasitism. The IL-4 and IL-13 stimuli also promoted an increase in parasitism associated with an increase in IGF-I expression and an increase in arginase activity. Silencing *Igf-I* mRNA using IGF-I siRNA, the IL-4 and IL-13 stimuli led to decreased parasitism compared with their controls without siRNA. These data suggested that in the infection by *L. amazonensis*, IGF-I is also needed to promote susceptibility to infection (Figure 7). However, the effect of IFN-*γ* and interplay with IGF-I on *L. amazonensis* proliferation need further studies.

Thus, the susceptibility and resistance observed in *L. major*- and *L. amazonensis*-infected mouse strains may be due to cytokines to some extent, but the susceptibility essentially depends on the presence of IGF-I.

## 6. IGF-I in Susceptible and Resistant Leishmaniasis Mouse Models

In light of our findings showing IGF-I ruling susceptibility and resistance to *L. major* infection in vitro, we proceeded to evaluate the participation of IGF-I in vivo in *L. major*-infected susceptible (BALB/c) and resistant (C57BL/6) mouse strains. In control BALB/c mice, the lesion continuously progressed as expected, while in those animals injected with parasites preincubated with IGF-I, the lesion development was accelerated, becoming larger when compared with the control. In contrast, in control C57BL/6 mice, the lesions progressed for three weeks and then stabilized and tended to diminish, but in those animals infected with parasites preincubated with IGF-I, the lesion interestingly progressed continuously, and it was significantly greater than that in the control, although smaller than that in the BALB/c mouse.

The previous data, showing that the transfer of BALB/c highly expressing IL-4 cells to genetically resistant chimeric mice on a C57BL/6 background did not result in susceptibility [15], suggested that the infection outcome is not governed only by the type of cytokine produced. Then, we asked whether IGF-I expression in mouse strains may explain the *L. major* infection outcome. The IGF-I expression in susceptible BALB/c mice and resistant C57BL/6 mice infected with *L. major* was indeed different. We evaluated the *Igf-I* mRNA by qPCR and IGF-I expression in anti-IGF-I labeled cells under confocal microscopy. By both approaches, IGF-I was detected in higher levels in BALB/c mice-derived peritoneal macrophages than in C57BL/6 mice-derived cells [65], suggesting that background expression of IGF-I may count determining susceptibility to *L. major* infection in mice.

Remarkably, in the *L. braziliensis*-infected mice, the disease development is not tangible, with tiny lesions [104]. The same is observed in a visceral leishmaniasis murine model using *L. donovani* or *L. infantum* that presents self-controlled disease [105, 106]. Coincidentally, the IGF-I in vitro effect on these *Leishmania* species was different from that observed with *L. major* and *L. amazonensis*. Whether these features are somehow related to IGF-I is an open question that deserves further studies.

## 7. IGF-I in Human Leishmaniasis

In human leishmaniases, the infection development is related to the host's genetic and immunological characteristics and the *Leishmania* species' characteristics. We should emphasize the differences presented here on the IGF-I effect on the infection development, depending on the *Leishmania* species. We should also consider the sizable differences between the diseases seen in human patients and murine models.

In murine CL, in the mouse strains susceptible to *L. majo*r and *L. amazonensis*, the lesions' development is progressive. In these lesions, we find macrophages full of *Leishmania* amastigotes in proliferation [105]. In human active CL caused by these *Leishmania* species, the lesion presents different chronic inflammatory processes and scanty amastigotes [106, 107]. Only in rare diffuse cutaneous leishmaniasis, caused by *L. amazonensis*, the lesions are in some way similar to those observed in the susceptible murine CL model with abundant amastigotes in the lesion [107].

We addressed the VL caused by *L. infantum* and tegumentary leishmaniasis caused by *L. braziliensis* to study the participation of IGF-I in human leishmaniases in Brazil.

### 7.1. IGF-I in Human Visceral Leishmaniasis

As shown above, the disease progression was initially attributed to *Leishmania* antigen-specific immunosuppression in active VL, but what we notice is an immune activation and imbalance of the immune response. Immunopathogenesis of VL is still not clear, and recently, CD8^+^ T cells were characterized by a gene signature observing the increased expression of specific cytolytic (granzymes A, B, and H and perforin), cytokine signaling (SOCS3, STAT1, JAK2, and JAK3), and immune checkpoint genes (LAG-3, TIM-3, and CTLA-4) [108].

When we evaluated both IGF-I and IGF-binding protein-3 (IGFBP3) serum levels in samples collected from active VL patients, we observed low levels of both IGF-I and IGFBP3 [109]. Based on these unexpected data, mainly in VL, where we observe massive parasite proliferation in inner organs and considering experimental data showing no evident IGF-I effect on parasite growth, we should consider the participation of IGF-I in another way in the biology of *Leishmania*. Further IGF-I low levels in active VL were also seen in canine VL [109, 110] and human VL patients [111]. We do not have any element to envisage whether an IGF-I low level seen in active VL cases influences nonspecific processes related to parasite growth or adaptive immune response.

Alternatively, considering IGF-I's pleiotropic effect, we regarded it as IGF-I participation in other aspects of disease pathogenesis. High levels of IFN-*γ* and TNF-*α* in active VL may explain low IGF-I serum levels [66, 112]. We noticed that IGF-I is linked to hematopoiesis and anemia [113, 114]. Both IGF-I and IGFBP3 serum levels showed a positive correlation with hemoglobin levels in active human VL and canine VL. Thus, we suggested that IGF-I has a pathogenic role in VL anemia without any correlation with cytokine levels. IFN-*γ* was shown negatively correlated with anemia; thus, we suggested that both IFN-*γ* and IGF-I contribute to the pathogenesis of anemia in active VL but independently [109]. The experimental data in murine VL showing CD4^+^ T cells producing IFN-*γ* with alteration in the stromal microenvironment in bone marrow linked to anemia development reinforced the importance of IFN-*γ* in the pathogenesis of anemia in active VL [115].

### 7.2. IGF-I in Human Tegumentary Leishmaniasis

Tegumentary leishmaniasis in Brazil is mostly caused by *L.* (*Viannia*) *braziliensis*, which presents as CL, ML, and DL. Studies on pathogenesis show that immune response participates not only in parasite growth control but also in lesion development [116]. In CL, there is a strong T cell response with Th1 cytokine production, such as IFN-*γ* and IL-12, related to infection control, but if uncontrolled, it may cause tissue damage [117]. The ML has a high specific T cell response, both Th1 and Th2, directed to a Th1-type response. High levels of proinflammatory cytokines, TNF-*α* and IFN-*γ*, are produced, which are poorly regulated by IL-10 and TGF-*β* [118, 119].

Further, comparing infected asymptomatic, CL, and ML cases, a higher level of TNF-*α* was seen in CL and ML lesions than in asymptomatic individuals. This higher level of TNF-*α* is involved in lesion development even though it has known anti-*Leishmania* effect [120]. Another study showed that both CD8^+^ T cells and granzyme were related to lesion development [121]. In a more recent study, transcriptomic analysis in skin samples compared gene expression in cured and noncured CL patients after 90 days of treatment. Gene sets related to cytolytic machinery were significantly more expressed, with higher expression of granzyme (*GZMB gene*), perforin (*PRF1 gene*), and granulysin (*GNLY gene*) [122] in noncured CL patients. Thinking on the participation of IGF-I in the pathogenesis of *L. braziliensis*-caused CL, the role of IGF-I would be more related to inflammatory and healing processes than to parasite growth.

We initially addressed IGF-I in human cases of leishmaniasis, measuring both IGF-I and IGFBP3 serum levels in patients presenting different clinical forms, CL, ML, and DL. In this analysis, both IGF-I and IGFBP3 levels were lower in ML and DL than CL and healthy controls [89]. Considering the pleiotropic effects of IGF-I and observing a low level of IGF-I in patients with worse clinical presentations such as ML and DL, we may speculate on IGF-I's role in the modulation of the inflammatory process and the maintenance of epidermis and healing process [123–127].

Searching the role of IGF-I by immunohistochemistry in the lesion of 51 human CL caused by *L. braziliensis*, IGF-I was seen related to chronicity and good response to treatment, but not parasite growth, and we relate the findings to the efficient anti-inflammatory response and the known action of IGF-I in wound repair [128].

## 8. Perspectives on the Use of IGF-I in Therapeutic Interventions in Human Leishmaniasis

IGF-I was associated with other skin diseases where delayed wound healing was related to IGF-I's low production at the injury sites. In conditions like diabetes mellitus, IGF-I has been used in skin ulcer treatment observing the healing with an increase in the IGF-I local level upon hyperbaric oxygen therapy or using IGF-I cream locally [129, 130].

Interference on the IGF-I pathway has been proposed to address treatment strategies in diseases such as cancer, autoimmune diseases, and atherosclerosis [131–133]. One of the suggested targets is the Th17/Treg axis. Th17 cells were associated with protection in VL but were associated with infiltration and disease pathology in human CL and ML [134]. In autoimmune disorders with the participation of Th17 cells, beneficial effects of a systemic recombinant IGF-I treatment were seen through an increase of regulatory T cell levels in affected tissues. Regulation of Th17 cells by IGF-I may occur through modulation of AKT-mTOR and STAT3 signalings [72]. Another target is the Vascular Endothelial Growth Factor A (VEGFA), a key factor in angiogenesis and wound healing process [135].

The possibility to explore the therapeutic use of IGF-I encourages us to proceed with the studies on IGF-I in the pathogenesis of different forms of leishmaniases.

## 9. Conclusions

In leishmaniases, because the adaptive immune response does not fully explain *Leishmania* infection's outcome, we addressed the participation of IGF-I in infection and disease outcome. Here, we reviewed the role of IGF-I in leishmaniasis experimental models and human patients. IGF-I's effect extends over the biology of *Leishmania*, *Leishmania*-macrophage interaction hitting arginine metabolic pathway in both cells, cytokine modulation, and pathogenic mechanisms of different disease manifestations. The direct effect of IGF-I on *Leishmania* results in its growth in vitro at a specific parasite stage. It influences the disease's course inducing an increase in the skin lesion size and parasite load, especially with *L. major-* and *L. amazonensis-*infected mouse cutaneous leishmaniasis. With other species of *Leishmania*, *L. braziliensis* and *L. infantum*, parasite growth is not evident, bringing question on the differences in arginine metabolic pathway activation dependent on the parasite species.

IGF-I interacts with cytokines where IFN-*γ* inhibits, while IL-4 and IL-13 increase its expression in macrophages. In the interaction with IL-4, a cytokine that is a hallmark of susceptibility to *L. major* in murine leishmaniasis, we show IGF-I as an effector element of the IL-4, an unprecedented finding.

Moving to human leishmaniasis, IGF-I was not proven as a factor promoting parasite growth in cutaneous leishmaniasis caused by *L. braziliensis* and visceral leishmaniasis by *L. infantum*. Since patients with more severe diseases such as mucosal, disseminated, and visceral forms presented low IGF-I serum levels, alternative roles were searched. We observed that low IGF-I levels might contribute to the inflammatory response persistence and delayed lesion healing in human cutaneous leishmaniasis and the anemia development in visceral leishmaniasis. We must highlight the complexity of infection revealed depending on the *Leishmania* species and the parasite's developmental stages. Because IGF-I exerts pleiotropic effects on the biology of interaction and disease pathogenesis and can trigger and/or modulate more than 200 genes in certain cells and tissues, IGF-I turns up an interesting tool to explore biological and pathogenic processes underlying infection development. Further, IGF-I pleiotropic effects open the possibility to approach IGF-I as a therapeutical target.
